# Novel Bionics Assessment of Anorectal Mechanosensory Physiology

**DOI:** 10.3390/bioengineering7040146

**Published:** 2020-11-14

**Authors:** Hans Gregersen

**Affiliations:** 1 The Chinese University of Hong Kong, Shatin, Hong Kong SAR, China; hag@giome.org; 2 California Medical Innovations Institute, San Diego, CA 92121, USA

**Keywords:** anorectal physiology, biomechatronics, bionics, colon transit, defecation, Fecobionics, functional testing, mechano-physiology, modeling, simulated stool

## Abstract

Biomechatronics (bionics) is an applied science that creates interdisciplinary bonds between biology and engineering. The lower gastrointestinal (GI) tract is difficult to study but has gained interest in recent decades from a bionics point of view. Ingestible capsules that record physiological variables during GI transit have been developed and used for detailed analysis of colon transit and motility. Recently, a simulated stool named Fecobionics was developed. It has the consistency and shape of normal stool. Fecobionics records a variety of parameters including pressures, bending, and shape changes. It has been used to study defecation patterns in large animals and humans, including patients with symptoms of obstructed defecation and fecal incontinence. Recently, it was applied in a canine colon model where it revealed patterns consistent with shallow waves originating from slow waves generated by the interstitial Cells of Cajal. Novel analysis such as the “rear-front” pressure diagram and quantification of defecation indices has been developed for Fecobionics. GI research has traditionally been based on experimental approaches. Mathematical modeling is a unique way to deal with the complexity. This paper describes the Fecobionics technology, related mechano-physiological modeling analyses, and outlines perspectives for future applications.

## 1. Introduction

Biomechatronics (often abbreviated bionics) is an applied science that aims to tie interdisciplinary knots between biology and engineering (mechanical, electrical, and electronics engineering). The term bionic was coined by Steele in 1958 and popularized in science fiction films by the movie industry with humans given supernatural powers by electromechanical implants. It mimics how the human body works. Bionics is probably best known in development of prosthetic limbs, vision aids, robotics, and neuroscience. The bionics term overlaps with electrical medicine.

The gastrointestinal (GI) tract has always imposed challenges for studies of its function. This is due to the difficult access to remote parts of the GI tract that basically is a long tube with multiple compartments. The control of GI function is complex due to the layered structure and neural regulation.

During the last decades, bionics advancements have facilitated design and development of devices that can be ingested. Ingestible capsules contain cameras (Pillcam), pressure and pH sensors (Smartpill), or magnets that can be tracked. This has provided an excellent opportunity to study remote parts of the GI tract without apparent disturbance of the function. A recently introduced technology is the magnetic tracking system, which can measure motility, direction of movement, and transit time from the stomach, the small bowel and the colon [1]. Colon anatomy is well suited for 3D modeling of transit. A recent analysis has been utilized for classification of different motility patterns based on the velocity and length of magnetic capsule movements [2,3,4]. Insight into colonic motility offers many possibilities for classification of dysmotility symptoms or interventional testing of pharmacological treatments. However, despite the clinical need for new technology for the many patients with lower GI tract symptoms, current technology is limited, e.g., the magnetic tracking system is not commercially available, and often results differ between the technologies in use [5].

Within the past years a simulated stool named Fecobionics with the shape and properties of normal stool was developed [6,7,8,9,10,11]. Fecobionics records, in a single experiment, numerous parameters including pressures, bending, and shape changes during colonic transport and defecation of the device. Fecobionics has been used to study defecation patterns in large animals as well as humans (normal subjects and select patients groups including patients with symptoms of obstructed defecation and fecal incontinence). Very recently, it was applied in a canine colon model for evaluation of neuromuscular function and transit patterns [12]. Novel analysis such as the rear-front pressure (preload-afterload) diagrams and quantification of defecation indices have been developed. Furthermore, papers have been published on modeling of Fecobionics data [10,11].

Modeling of GI function is becoming more important than ever and is necessary for deeper understanding of the mechanical aspects or organ function, e.g., to predict progression of diseases. Advanced high-resolution clinical measurement technologies and rapid advances in computational power now allow construction of comprehensive mechanical models of GI organs [13,14,15,16,17,18,19,20].

This paper briefly reviews the Fecobionics technology including modeling studies, and outlines perspectives for future applications.

## 2. Lower GI Tract Physiology and Functional Disorders

Colon serves to absorb water and salts from luminal content and pushes the content towards the rectum where it is stored before defecation. Normal colonic function depends on complex mechanisms involving intestinal neuromuscular circuits as well as signaling from the central nervous system. Various colonic contraction patterns have been identified [21]. The final transport path in the GI tract is the expulsion of fecal contents from the rectum. Anal continence and defecation involve anatomical factors, anorectal sensation, rectal compliance, stool consistency, anal muscle strength, mobility, and psychological factors [22]. In contrast to the intestines, the pelvic floor muscles are under conscious control. The homeostatic balance is easily disturbed by functional or structural anorectal disturbances that may coexist.

Defecation is the physiological process through which stools are eliminated from the rectum via the anal canal [23,24,25]. Defecation is initiated by urge to defecate predominantly resulting from filling of stool in the rectum. The abdominal pressure increases during defecation, the anal sphincter and puborectalis muscle relax, and the anorectal angle straightens [24,25,26]. The defecation process may be disordered, resulting in symptoms such as fecal incontinence, constipation, and pain (proctalgia) [22]. Colonic and defecatory disorders affect 25% of the population with rising incidence [22,23,27,28]. These disorders pose a major health care burden but are poorly recognized and treated [22]. For example, constipation refers to abnormally delayed or infrequent passage of usually dry hardened feces that may be associated with pain during defecation. Chronic constipation (CC) is a symptom of possibly several underlying pathophysiologic processes and affects 12–19% of Americans [29]. The etiology of CC is multifactorial. Slow colonic transit and anorectal disorders are major causes of CC. Anorectal sensitivity and contractility, stool consistency, rectal reservoir capacity and compliance, and coordination of the pelvic floor muscles play important role in the genesis of obstructed defecation. Etiology of slow colonic transit constipation includes low residual diet, female sex hormones, medication that inhibit peristalsis and coordination (such as opioids and anticholinergics), and neuromuscular disorders of the colonic smooth muscles.

There are many tests available currently to study anorectal motility disorders but it is not clear whether the tests identify the precise pathophysiologic abnormality. Commonly used tests are high-resolution anal manometry (HRAM), barium and MR defecography, balloon expulsion test (BET), and barostat testing for the rectal sensory function [22,26,30,31,32,33]. These tests have significant limitations; e.g., HRAM does not record dynamic events during real defecation, defecography does not provide information on anorectal sensation and motility, and BET does not provide physiologic information; i.e., geometry and pressure changes during the passage of simulated stool from the rectum through the anal canal. Furthermore, there is considerable disagreement between the results of various anorectal tests and not surprisingly the test results correlate poorly with symptoms and treatment outcomes [5]. Most importantly, there is no integrated diagnostic test to define the anorectal dysfunction in obstructed defecation. The need for physiologically relevant and easy-to-use diagnostic tests for identifying underlying mechanisms is substantial.

Colon is more difficult to study than anorectum due to the remote access and lack of technology. Ingestible markers visible by radiography or magnetic sensors have been used for years to study colonic transit. This is important for diagnosis of slow transit constipation but has little value for studying irritable bowel syndrome. The recent development of fiber-optic high-resolution manometry catheters with more than 72 pressure sensors has advanced the field. Recordings have been made of motor events in the entire colon over prolonged periods of time [34,35]. Various studies describe patterns of colonic motor events including high amplitude peristaltic contractions and retrograde movement of markers [21,36]. However, the abovementioned in vivo technologies have not been able to detect the shallow waves observed in colon preparations from rabbits or rodents in vitro [21]. These shallow waves are believed to play an important function for slowing colonic transit and for mixing [21].

## 3. Design Considerations for Fecobionics and Principle of Measurement

To be a truly bionics device for colon and anorectal transit studies, the shape and consistency of Fecobionics must be like feces and measure relevant physiological data. Indeed, the shape and properties of Fecobionics were designed to be similar to the most common form of feces in normal subjects according to the Bristol stool form scale [37]. Feces consistency changes along colon and a choice has to be made which part of colon to simulate best since feces becomes more solid during the transit. Colon and anorectal transport of feces depends on several factors that are desirable to measure. The most relevant parameters are the pressures (forces) acting on the surface of feces, the size of the intestinal conduit, friction between feces and the intestinal mucosa, and bends of the conduit. Axial pressure in the direction of the trajectory is of particular interest. Distensibility and sensitivity are other parameters of importance. All parameters must be considered in design of a bionics device for simulation of GI transport. Fecobionics is conveniently designed to quantitate the mentioned parameters. The most important variables are briefly discussed below for anorectal applications. Derived parameters are discussed in the section on detailed analysis and modeling.

### 3.1. Anorectal Pressures 

Defecation is a mechanical process where feces in the rectum is expelled through the anal canal. Typically, when a person feels the urge (due to activation of mechanoreceptors in the rectum), he or she move to the toilet or another appropriate place. The rectal or abdominal pressure is increased with simultaneous relaxation of the puborectalis muscle and anal sphincter. Since feces is transported inside the anorectal conduit, we must measure the forces (pressures) that push it forward and expel it as well as measure the resistance to the flow in the anal canal. In order to measure these pressures, Fecobionics has pressure sensors embedded at the front and rear of the probe, pointing in the direction of the trajectory. This is a major novelty compared to existing technology and important because pressure is directional. Current technology such as HRAM measures radial pressures, which are distinctly different from the pressures recorded by Fecobionics. The rear pressure in Fecobionics is a proxy of the expulsion force (analog to cardiac preload) whereas the front pressure measures the anal pressure that oppose the pushing force (analog to cardiovascular afterload). The anal sphincters are the major contributor to the anal high-pressure zone and therefore the main contributor to resistance to flow. Furthermore, it is of interest to measure the pressure inside the distending bag [24,38] since it is a proxy of the force applied to the intestinal wall and can be used to compute tension [24,38]. Fecobionics has a pressure sensor mounted in the middle of the probe inside the bag.

### 3.2. Bending 

Another contributing factor to the resistance to flow and continence function is the angulation between the rectum and the anal canal. This is termed the anorectal angle [39]. It is caused by tonic activity of the puborectalis muscle that encircles the upper anal canal [40]. The anorectal angle is approximately 120° during resting conditions and straightens towards 180° during defecation [39,41]. Fecobionics provides an electronic measure of its own bending, which is a proxy of the anorectal angle when Fecobionics is being defecated. Current technology is defecography where the anorectal angle is measured from radiographic images.

### 3.3. Dimensions 

Transport in luminal organs depend not only on the pressure difference but also on luminal dimensions and geometry. For example, the diameter and length of the anal canal have a large impact on anal resistance during defecation [24,38]. It is important to know the rectal diameter since the rectum acts as a reservoir with stool accumulation before defecation. Dimensional data provide information on organ deformation. Dimensional measures including the organ radius are useful for computation of tension and elastic moduli [38]. Fecobionics quantitates cross-sectional areas using the impedance planimetry principle and shape and simultaneously measures the bag pressure. Cross-sectional areas are estimated from measurements of electrical impedance of the saline between adjacent electrodes inside the bag [42].

### 3.4. Sensation 

Sensation is closely linked to change in dimensions since mechanosensitive receptors are widely believed to be stretch sensitive. In the rectum, sacral dorsal roots nerves contain afferents from low-threshold mechanoreceptors located in the rectal wall. These receptors monitor the filling state and the contraction level of the rectum [43]. Anal sensation is carried out by several types of receptors. At rest, the aforementioned factors keep stool within the rectum. When distended to a certain level, mechanoreceptors are stimulated and initiate defecation urge. Rectum distension causes the internal sphincter to relax. The abdominal muscle contraction overcomes the resistance of the anal sphincters and the pelvic floor descends during defecation. As mentioned above, Fecobionics measures bag dimensions and pressure. Therefore, tension and deformation can be assessed, which is useful for analysis of sensory responses [7,11,24,38].

### 3.5. Fecobionics Design 

**Figure 1:**
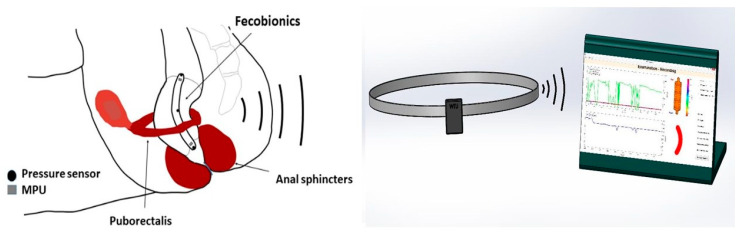
The Fecobionics system with device shown inside the rectum, the wireless transmitter, and the computer with the graphical user interface.

**Figure 2:**
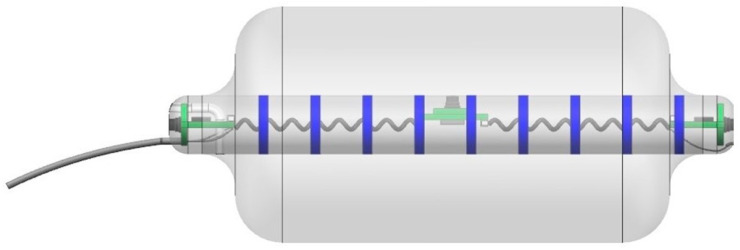
The newest wireless version of Fecobionics with pressure sensors, motion processing units, impedance electrodes, CPU, wireless transmitter and six internal batteries. The tube to the left is detachable. Dr. Daming Sun is thanked for the animation of the device.

Fecobionics is a simulated stool capable of dynamic measurements in the lower GI tract [6,7,8,9,10,11]. It was originally developed to simulate the defecation process where it integrates several current technologies in a single examination. The basic design of Fecobionics has been described and the newest wireless design is sketched in Figure 1 and Figure 2. In brief, Fecobionics is an elongated flexible probe made from silicone. Silicone is the ideal core material due to its softness, durability, non-degradability, and electrical current insulation. The silicone core insulates the embedded electrical components from being in direct contact with tissue. This is important if batteries leak or the electronics short-circuits. The hardness of the silicone core can be changed to provide different types of fecal consistency.

The Fecobionics probe length is 10–12 cm and the outer diameter is 10–13 mm. Most of the length is spanned by a bag that can be filled up with saline. Fecobionics with the bag was designed to have consistency that corresponds approximately to type 4 (range 3–4) on the Bristol stool form scale. The range from types 3–4 is found in +60% of healthy subjects [37]. The bag is connected through a thin tube extending from the front of Fecobionics to a syringe containing saline. In newer versions, the filling tube is detachable.

The electronic sensors in Fecobionics are three pressure sensors, two motion processing units (MPUs) and impedance electrodes for measurement of the cross-sectional area (CSA) and shape of the fluid-filled bag [6]. Furthermore, the probe contains the MicroControl Unit (MCU) that processes the data from the sensors. In wired versions, wires are threaded inside a thin tube extending from the front to the USB port of a computer for power supply and for real-time data transmission. Data are displayed in real time on the graphical user interface. Newer wireless versions of Fecobionics contain batteries and a wireless radiofrequency transmitter (Figure 1 and Figure 2).

The wired Fecobionics contained 6-axis MPUs with gyroscopes and accelerometers for orientation measurements [6]. Newer versions contain 9-axis MPUs with magnetometers in addition to gyroscopes and accelerometers. This allows for improved computations based on less assumptions. The bending angle is computed from data from the two MPUs placed at the rear and front of Fecobionics using the Madgwick algorithm [6,44]. The impedance electrodes mounted on the surface of the core inside the fluid-filled bag are used to measure 7–8 adjacent CSAs. Using the impedance planimetric principle, two outer electrodes generate a constant alternating electrical field inside the saline-filled distension bag. Multiple equidistant detection electrodes measure the impedance of the fluid between them, which can be calibrated to CSAs. The bag shape can be computed from the serial CSAs. Technological validation for the original wired Fecobionics have been published [6].

## 4. Fecobionics Assessment of Functional Data

**Figure 3:**
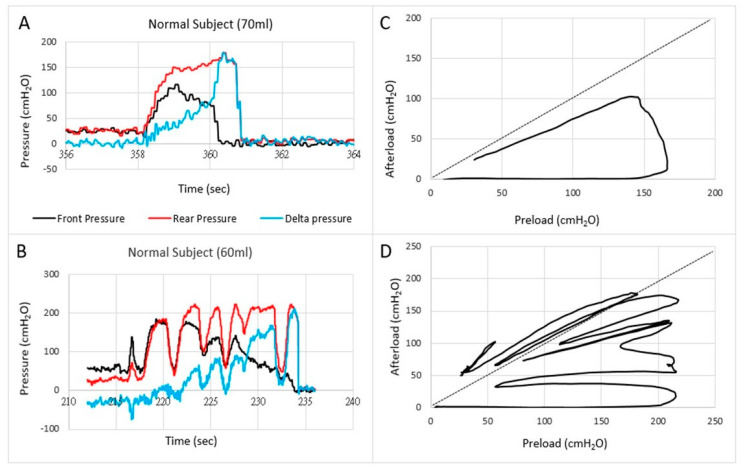
Examples of pressures as a function of time (**A**,**B**) and the front pressure plotted as a function of the rear pressure in two normal subjects (**C**,**D**). The first contraction usually follows the line of pressure unity where after the front pressure progressively decreases due to anal sphincter relaxation. In some subjects, this happened in one contraction (**C**) whereas others use several contractions (**D**).

Anorectal Fecobionics data have been published on normal human subjects as well as in a small number of patients with fecal incontinence and constipation [7,8,9]. Most attention has until now been given to pressure analysis. Typically, the front, rear, and bag pressures, as well as the difference between front and rear pressures are plotted as function of time (Figure 3). Endpoints such as the resting and maximum pressures can be derived from the plots. Based on pressure-time plots, it was possible to divide defecations into five phases based on distinct pressure patterns [7]. Rear-front pressure (preload-afterload) plots are another informative way to express pressure data. The preload-afterload concept is known from cardiology, where left ventricle pressure-volume measurements provide substantial insights into heart contractility, preload (heart filling), and afterload (vascular resistance) [24,38,45]. This type of analysis is beneficial for understanding anorectal function.

Translation of the learnings from cardiology to gastroenterology for Fecobionics testing, the filling of the bag inside the rectum until the subject feels the urge, corresponds to the preload. At this point, the subject initiates abdominal contractions to generate the propulsive force needed to expel the device. The driving force is picked up by the rear pressure sensor whereas the front pressure sensor records the afterload. The diagram allows evaluation of pressure cycles without the time element. When the front pressure exceeds rear pressure, data are above the unity line (defecation cannot happen against a pressure gradient). Fecobionics (and feces) will be expelled when the recto-anal pressure gradient is large enough to overcome the friction between the surface and mucosa. Measurement of axial pressures at front and rear, and the bag pressure is essential in this regard. Repeated contractions shift the tracings downwards where a cut-off is reached, i.e., the anal pressure drops quickly followed by device expulsion [7,8,9]. Afterload is the resistance that the propulsive force must work against to evacuate feces. The resistance depends on several factors including anal diameter, anal pressure, anorectal angle, and friction. The diagrams show clockwise contraction cycles, for normal subjects usually 2–5 cycles, reflecting the number of abdominal muscle contractions that are needed to defecate. Fecobionics is uniquely designed to quantify these preload-afterload properties. Figure 3 shows representative defecations from two normal subjects. For comparison, fecal incontinence patients are often below the unity line and defecate instantly. In contrast, constipation patients are often above the line of unity for an extended time and use multiple contraction attempts.

The preload–afterload diagrams are very intuitive but must be quantified. Therefore, defecation indices are computed as the areas under the front pressure curve (reflecting anal resistance) and rear pressure curve (reflecting propulsive force). The defecation indices can be normalized with respect to duration, maximum pressure amplitude, as well as other factors. The general learnings from yet unpublished studies is that fecal incontinence patients have a low index whereas constipation patients have high index. Data points to that subtypes exist both for fecal incontinence and constipation patients. The afterload seems especially important since obstructed (dyssynergic) defecation [46,47,48] and anal stricture will be associated with increased afterload. The preload and afterload may be clinically important for differentiating subtypes of patients. For example, the current dyssynergia classification [46,47,48] uses a 2 × 2 diagram where two subtypes show abnormal expulsion pressures and two subtypes are associated with anal sphincter function. The classification is being criticized for being too simple and dyssynergic abnormality has been found in 90% of healthy subjects with HRAM [49]. Preliminary data with Fecobionics point to that more than four subtypes exist. This needs further investigation.

**Figure 4:**
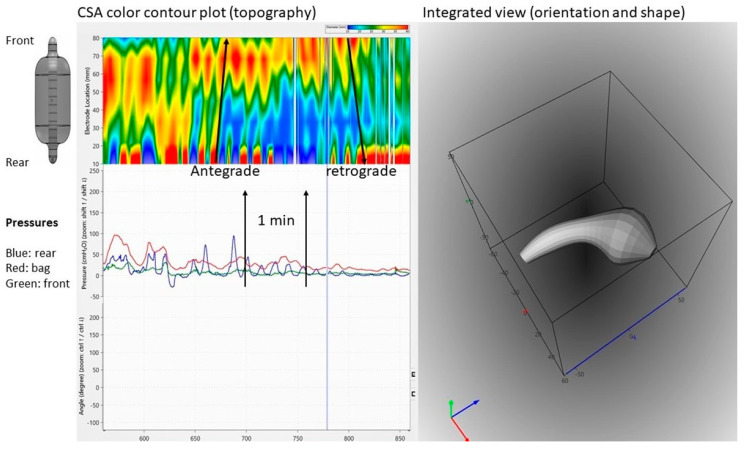
Graphical user interface showing data recordings from the canine colon. Upper left: Color contour plot generated from multiple cross-sectional area (CSA) recordings demonstrating antegrade and retrograde shallow waves that are not recorded by manometry. Bottom left: Pressure recordings from the front, bag, and rear. Right: 3D plot of the orientation, shape, and bending of Fecobionics (at the time point indicated by the vertical marker in the left diagrams.

Fecobionics has recently been applied to the colon in a canine study [12]. From in vitro experiments in rodents and rabbits, it was well known that shallow contractions (ripples) exist [21]. These were recorded as subtle diameter changes detected by video capture. However, such waves are not detectable with manometry. Fecobionics was able to pick up shallow antegrade and retrograde contractions in the canine colon, as well as antegrade peristaltic contractions. The shallow contractions are believed to serve an important function for slowing down colonic transit and facilitate mixing of fecal content. Figure 4 shows an example of shallow contractions in the transverse colon. The color contour topography plot, originally developed by Clouse for high-resolution manometry, is employed [50].

## 5. Advanced Analysis and Modeling

Modeling of lower GI tract functional data including anorectal data is still in its infancy. This is unfortunate since it is evident how well computational modeling has benefitted our understanding of other organs. From a bioengineering perspective, modeling efforts should first focus on construction of anatomically correct models, mechano-physiological models, and finally mechanosensory models can be advanced [13,24,38,51,52,53,54,55]. Anorectal models have been proposed [25,56,57] and are currently based on flow equations that are relatively simple for describing defecatory function. The purpose of mathematical modeling is to be able to predict the occurrence/non-occurrence of the event in question. In order to build a successful model, it is critical to know all the variables or parameters involved in the occurrence of any event. Defecation is basically the motion of liquid/solid material from the rectum across the anal canal to the outside. Fecobionics provides relevant data of the defecation process for modeling. With the latest integrated version of Fecobionics technology, more solid modeling work can take place.

The CSAs along with the pressures at various locations facilitate calculations of tension, stress, strain, and elastic modulus. These properties are important for understanding the mechanosensitive receptors in the rectal wall and sensory responses to distension. Specifically, combining the geometric simulation with the force and deformation computations will provide better understanding of the defecatory process. Novel parameters such as frictional force and dynamic viscosity computations can be implemented in models of defecation [10,11].

Based on the mechanical analysis on the curved thin-walled shells, Liao and coauthors recently developed a mechano-physiological model of the anorectal system based on Fecobionics measurements [10]. Using the proposed model analysis, mechanical factors associated with defecation of the device, including the friction force between the anorectal wall and the device, longitudinal and circumferential tensions, expulsion velocity and dynamic distensibility of the anorectal system were successfully obtained [10,11].

The membrane tension analysis assumes that the Fecobionics bag and anorectal shape are curved thin-wall shells of revolution during the device movement. The anorectal wall resists the pressure induced by the distension and the shear stress through the friction between the bag and tissue wall. Using an arbitrary surface coordinate system, the following equations of equilibrium on the anorectal surface were derived:

(1) $$\alpha_{s}\frac{\partial \left(N_{\theta }\right)}{\partial \theta }+\frac{\partial \left(\alpha_{\theta }N_{s\theta }\right)}{\partial s}+\frac{\partial \alpha_{\theta }}{\partial s}N_{s\theta }-\frac{{d\alpha }_{s}}{d\theta }\left(N_{s}-N_{\theta }\right)=-\alpha_{s}\cdot \alpha_{\theta }\cdot f_{\theta }$$

(2) $$\alpha_{s}\frac{\partial \left(N_{\theta }\right)}{\partial \theta }+\frac{\partial \left(\alpha_{\theta }N_{s\theta }\right)}{\partial s}+2\frac{\partial \alpha_{s}}{\partial \theta }N_{s\theta }-\frac{{d\alpha }_{\theta }}{\mathrm{ds}}N_{\theta }=-\alpha_{s}\cdot \alpha_{\theta }\cdot f_{s}$$

(3) $$\frac{N_{s}}{R_{s}}+\frac{N_{\theta }}{R_{\theta }}=-f_{w}$$

where $f_{w}$ is the force across the tissue wall, $f_{s}$ and $f_{\theta }$ are the distributed force (force per unit area) between Fecobionics and the wall along the s and θ directions. Membrane stress resultants were $N_{s}$, $N_{s\theta }$, and $N_{\theta }$, the longitudinal, shear, and circumferential membrane tensions, respectively. Details about the definitions of these coordinates and geometric features have been published [10]. The boundary condition for the bag in anorectum is $N_{s}=N_{s\theta }=0$ when s→∞. During defecation, the distension, contractile force from the anorectal muscles, and forces from the probe will cause wall deformation. The forces across the wall were assumed representative of the recorded bag pressure. Hence, the membrane tensions during Fecobionics expulsion can be determined from the equation.

For Fecobionics to be expelled through the anorectum, a variety of forces acting on it must be overcome. According to Newton’s second law, the friction force between the surface and the wall ($F_{f}$) during Fecobionics expulsion can be calculated with an estimated expulsion velocity along the anorectal segment as:

(4) $$\sum^{\;}F_{s}=\left[\left(-P_{r}+P_{b}\right)\cdot A_{rear}+\left(P_{f}-P_{b}\right)\cdot A_{front}\right]+F_{f}-G_{s}=m\dot{v}$$

(5) $$F_{f}=m\dot{v}-\left\{\left[\left(-P_{r}+P_{b}\right)\cdot A_{rear}+\left(P_{f}-P_{b}\right).A_{front}\right]-G_{s}\right\}$$

where $P_{b}$ is the bag pressure. $P_{r}$ and $P_{f}$ are rear and front pressures. $G_{s}$ is the gravity component in the *s* direction, $A_{rear}$ and $A_{front}$ are the surface areas at both ends of the probe, *m* is the mass, $\sum^{\;}F_{s}$ is the resultant force along the *s* direction. $\dot{v}=dv/dt$ is the acceleration of the movement, $v=dl_{AA^{’}}/dt$ is the probe expulsion velocity. The velocity is assumed v=0 before the front starts to slide into the anal canal. Hence, the force distribution (force per unit area, $f_{s}$) in the *s* direction between Fecobionics and the anorectal wall is determined as:

(6) $$f_{s}\;=\;\frac{\sum^{\;}F_{s}}{A_{surface}}$$

where $A_{surface}$ is the contact surface area between the probe and the anorectal wall. The distribution forces along and normal to the *s* direction were assumed symmetrically loaded to the center line of the anorectal surface. Hence, the shear membrane tension $N_{s\theta }$ is eliminated. More comprehensive models are currently under development.

## 6. Perspectives

Fecobionics is a novel technology for assessment of colonic and anorectal physiology. The device is a simulated stool, capable of dynamic measurements of a variety of variables during colonic transport and defecation in a single examination. The data facilitate novel analysis of lower GI tract function as well as providing the foundation for modeling studies. Advanced analysis can enhance our physiological understanding of defecation and future interdisciplinary research for unraveling colonic transit, defecatory function, anorectal sensory-motor disorders, and symptoms. This is a step in the direction of improved diagnosis of anorectal diseases.

Future physiological and clinical studies may require design changes. The bending stiffness of the device can be changed by altering the silicone resin. This is important for studying subtypes of constipation associated with hard stools. Design changes of the graphical user interface may also be required for potential future use as biofeedback therapy at the point-of-care in the home of patients. The graphical user interface can inform the patients about correctly or incorrectly performed therapeutic maneuvers and ultimately health care personnel can be connected remotely in real-time to instruct the patient. Furthermore, other sensors such as EMG electrodes may be added in the device. Finally, Fecobionics can potentially be tracked in the colon. Fecobionics has embedded magnetometers. These magnetometers can be used for distance estimation using a magnet field generated from an external coil. Hereby, the position can be determined by triangulation.

A better understanding of colonic and anorectal function is important for the understanding of lower GI tract functional disorders such as slow transit constipation and dyssynergic defecation where the latter is believed to be the result of pelvic floor dysfunction [22,46,47,48,49]. Improved integrated diagnostics may aid individualized treatment of subtyped patients and define those who may benefit from biofeedback training [22]. As mentioned above, continence and normal defecation function depend on a variety of parameters that, too a large extent, are measured by Fecobionics in a single examination. The Fecobionics signature may provide clinically useful information for differentiation of physiology from pathophysiology. The clinical future of Fecobionics ultimately depends on its ability to impact on treatment of colonic and anorectal disorders. Potential clinical applications are diagnosis of slow transit constipation and diagnosis and biofeedback training for fecal incontinence and dyssynergic defecation. Fecobionics phenotypes and defecation indices show promise in physiological and diagnostic studies. However, parameters that may potentially change management of patients with functional anorectal disorders are yet to be defined. Furthermore, more comparative studies between Fecobionics and conventional technologies including HRAM, BET, and defecography are needed [9,11].

Computational modeling is a unique way to deal with complex problems in medicine. Based on the segmental anatomy and integrated mechanical, physiological, and pathophysiological characteristics of GI organs, the function can be quantitatively modeled. With the development of medical imaging techniques and advanced medical devices such as magnetic tracking of capsules and Fecobionics, modeling simulation methods can be integrated into standards of clinical work and ultimately aid to clinical decision making in gastroenterology.
